# Isolation of Dual-Active Drugs with Anticancer and Antibacterial Activities That Target Both Tubulin and FtsZ

**DOI:** 10.3390/antibiotics14101014

**Published:** 2025-10-13

**Authors:** Yanting Wang, Xufang Wang, Chunmeng Yao, Yaliang Zhang, Lantian Liu, Yan Cao, Bin Lu

**Affiliations:** 1 Department of Biochemical Pharmacy, School of Pharmacy, Second Military Medical University/Naval Medical University, Shanghai 200433, China; xufangwang@smmu.edu.cn (X.W.); yaochunmeng@smmu.edu.cn (C.Y.); lliu0136@student.monash.edu (L.L.); 2 State Key Laboratory of Pharmaceutical Biotechnology, School of Life Sciences, Nanjing University, Nanjing 210023, China; zhangyaliang@nju.edu.cn; 3 Faculty of Pharmacy and Pharmaceutical Sciences, Monash University, Melbourne, VIC 3052, Australia

**Keywords:** FtsZ, tubulin inhibitors library, FtsZ polymerization, SPR, molecular docking

## Abstract

**Background**: Cancer patients experience a high incidence of concomitant infections due to the effects of chemotherapy drugs and their suppressed immune function. Infection has become a major cause and an accelerating factor of cancer-related deaths. The combined use of anticancer drugs and antibiotics can produce adverse effects, necessitating the urgent search for dual-active drugs that are effective against both cancer and bacteria. Since tubulin has a homologous protein filamenting temperature-sensitive mutant Z (FtsZ) in bacteria, tubulin inhibitors have the potential to emerge as dual-active drugs against both cancer and bacteria. **Methods**: A comprehensive screening of a tubulin inhibitor library, encompassing 196 compounds, was conducted to evaluate their various activities. **Results:** Compounds **6**, **23**, **33**, **56**, **60,** and **71** exhibited both anticancer and antibacterial activities in vitro, and **23**, **33**, **56,** and **60** displayed varying degrees of FtsZ inhibitory activity. Particularly, compound **23** stood out as the most potent, exhibiting not only the strongest anticancer activity with IC_50_ values of 12, 20, and 10 nM against A549, MCF-7 and Hela cells, respectively, but also the most exceptional antibacterial activity with minimum inhibitory concentration (MIC) values of 8, 8, 64, and 32 μM against *Staphylococcus aureus* (*S. aureus*), *Bacillus subtilis* (*B. subtilis*), *Escherichia coli* (*E. coli*), and *Pseudomonas aeruginosa* (*P. aeruginosa*), respectively. Furthermore, compound **23** possessed the superior FtsZ inhibitory activity, facilitating polymerization. This was evident in the remarkably elongated cell morphology of *Bacillus subtilis* treated with compound **23**. To gain a deeper understanding of the underlying mechanisms, molecular docking studies were conducted, revealing the interaction mode between compound **23** and both tubulin and FtsZ, further elucidating its multifaceted biological activities. **Conclusions**: The dual-active drugs obtained in this study provide a new solution to the problem of bacterial infection in cancer patients. The revealed FtsZ as the antibacterial target provides an important theoretical basis for further optimization of such drugs.

## 1. Introduction

Cancer, as a systemic wasting disease with a high mortality rate, seriously threatens human life and health, and is a focal disease that needs attention in the world today [1]. Cancer patients exhibit a significant susceptibility to bacterial infections due to the effects of chemotherapy drugs and weakened immune function. During chemotherapy, infections are more common in the respiratory tract, oral cavity, gastrointestinal tract, and perianal area, leading to increased pain and decreased treatment compliance [2,3,4,5,6]. Infection is therefore the main cause and promoting factor of cancer death [7,8,9,10]. Consequently, bacterial infection in cancer patients is a crucial issue that urgently needs attention and resolution.

Both bacterial-infected and uninfected cancer patients require interventional treatment with antibiotics [11], so it is extremely common to employ both anticancer drugs and antibiotics simultaneously. However, the simultaneous use of anticancer drugs and antibiotics can produce various pharmacological and clinical effects, which can alter the therapeutic effects of each other and also affect their toxic effects [12,13]. There are cases showing that when chemotherapy drugs are used in combination with antibiotics, symptoms such as fever and decreased appetite may occur; stop using antibiotics gradually and use only anti-tumor drugs. The condition improves rapidly, and the body temperature drops to normal [12,13]. Given the potential drawbacks of combining anticancer and antibacterial drugs, there is an urgent need to develop drugs with both anticancer and antibacterial activities and low toxicity.

Microtubulin is a highly conserved GTPase family protein in eukaryotes, which is a key component of the cytoskeleton of cells [14,15,16]. Microtubulin inhibitors are a class of molecular targeted drugs that can achieve anticancer effects by inhibiting the polymerization or depolymerization of microtubules, such as paclitaxel, docetaxel, vincristine and ebomycin [17]. Additionally, a very important characteristic of microtubule proteins is the presence of their homologous protein filamenting temperature-sensitive mutant Z (FtsZ) in bacteria. The vast majority of bacteria and many archaea encode the FtsZ protein, which plays a central role in the cell division of most bacteria and many archaea [18]. FtsZ aggregates into filaments in a GTP-dependent manner, which assemble into a highly dynamic structure called the Z-ring on the inner membrane in the middle of the cell. As other cell division proteins recruit, the Z-ring contracts, leading to division. The inactivation of FtsZ leads to the loss of membrane formation, affecting bacterial cell division. Both FtsZ and microtubule proteins undergo GTP hydrolysis-dependent polymerization and depolymerization cycles [19,20]. In addition, although FtsZ and microtubule proteins have weak sequence similarity (<20%), they have broad functional similarities, and their crystal structures have very clear homology [21]. Therefore, compounds with strong affinity for microtubule proteins may also have strong affinity for FtsZ, meaning compounds with microtubule inhibitory activity may also have FtsZ inhibitory activity. Given that numerous microtubule inhibitors have been reported, but there has been no research on the relationship between microtubule inhibitors and FtsZ inhibitors, it is highly promising to screen the antibacterial activity of the already obtained or reported microtubule inhibitors to obtain inhibitors that combine anti-microtubule proteins and anti-FtsZ, namely anticancer and antibacterial dual-active compounds.

In this comprehensive study, we have undertaken a multifaceted approach to investigate the antibacterial potential of tubulin inhibitors. Specifically, our work encompasses the following key aspects. (1) Compound Library Construction: This study constructs a compound library containing 196 compounds. These compounds are mostly microtubule inhibitors with irregular structures. **1**–**81** have been reported in the literature, **82** is berberine, and **83**–**196** were designed and synthesized in the laboratory. (2) Antibacterial Screening of Tubulin Inhibitors: We have screened a diverse range of tubulin inhibitors, both sourced from literature reports and our own laboratory’s previous accumulations, for their potential antibacterial activity. This screening aimed to identify antibacterial agents within this class of compounds. (3) Affinity Estimation Using Biochemical Assays: To quantify the interaction strength between the identified compounds and their bacterial targets, we utilized two powerful techniques—FtsZ polymerization assays and Surface Plasmon Resonance (SPR) detection. These methods allowed us to accurately estimate the affinity between the proteins and compounds, providing crucial insights into their mechanism of action. (4) Molecular Docking Simulations: To gain a deeper understanding of the molecular interactions at play, we employed computer-aided molecular docking simulations. These simulations enabled us to visualize the interaction mode between the compounds and their protein targets, shedding light on the structural basis for their antibacterial activity and facilitating the rational design of more potent inhibitors.

## 2. Results

### 2.1. Minimum Inhibitory Concentration (MIC) Detection

The antibacterial activity of compounds was estimated, and the outcomes presented in Table 1 indicated that over half of the tested compounds exhibited robust antibacterial activity. Among these compounds, compound **71** stood out with the most potent antibacterial activity, with MIC values of 2, 32, 64, and 64 μM for *Staphylococcus aureus* (*S. aureus*), *Bacillus subtilis* (*B. subtilis*), *Escherichia coli* (*E. coli*), and *Pseudomonas aeruginosa* (*P. aeruginosa*), respectively. Additionally, compound **6** (MIC = 8, 16, 64, 32 μM), **23** (MIC = 8, 8, 64, 32 μM), **33** (MIC = 4, 64, 32, 2 μM), **56** (MIC = 128, 64, 128, 32 μM), **60** (MIC = 8, 64, 16, 8 μM), and **82** (MIC = 4, 64, 64, 8 μM) also demonstrated effective antibacterial activity. Conversely, the remaining compounds in the library failed to show obvious antibacterial activity. Therefore, seven compounds (**6**, **23**, **33**, **56**, **60**, **71,** and **82**) were selected for further investigation. It is noteworthy that although compound 6 exhibited MIC values in the micromolar range, its high molecular weight and limited solubility suggest that its apparent activity may be constrained by solubility. Its true target binding affinity might be higher than indicated by the MIC value.

### 2.2. Time–Killing Curve Determinations

**Figure 1:**
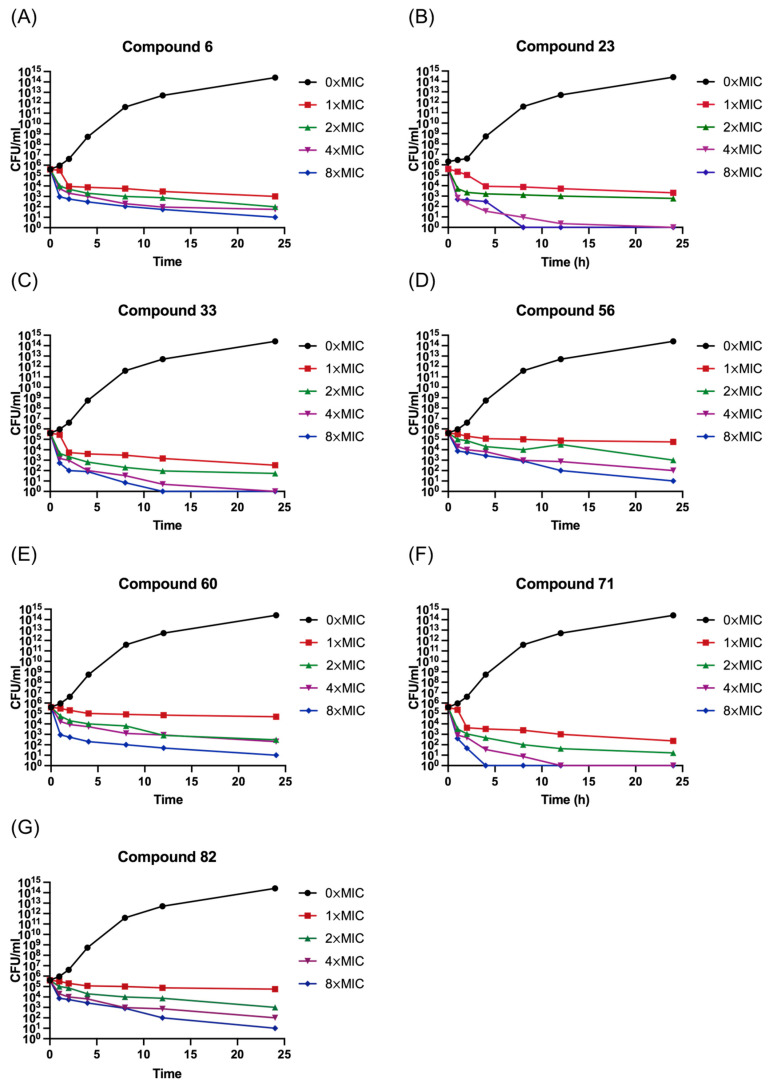
Time–killing curve of compound **6** (**A**), **23** (**B**), **33** (**C**), **56** (**D**), **60** (**E**), **71** (**F**), and **82** (**G**) against *S. aureus* BNCC336423. The different concentration of compounds was represented by different colors: 0 × MIC (1% DMSO) (black), 1 × MIC (red), 2 × MIC (green), 4 × MIC (purple), 8 × MIC (blue).

To more deeply investigate the bactericidal capabilities, a time–killing assay was performed at concentrations ranging from 1× to 8× MIC. By counting bacterial colonies at various time intervals, time–killing curves were generated to ascertain whether the compounds were bactericidal or not. The results of time–killing curves from compounds **6**, **23**, **33**, **56**, **60**, **71,** and **82** against *S. aureus* BNCC336423 are described in Figure 1. All tested compounds exhibited a clear bactericidal effect, the potency of which was dependent on both concentration and exposure time. This is a highly desirable trait for antibacterial agents, suggesting their efficacy can be optimized by dosing. Specifically, compounds **23**, **33,** and **71** achieved a rapid and significant reduction in viable count at this low concentration. Inspection of these time–kill data reveals that compounds **23**, **33,** and **71** produce 3 logs of kill at concentrations of 4× the MIC or greater. It is also of interest to note that at concentrations of ≥4× the MIC, the killing kinetics of compounds **23**, **33**, and **71** are more rapid than other compounds.

### 2.3. Sa-FtsZ Expression and Purification

**Figure 2:**
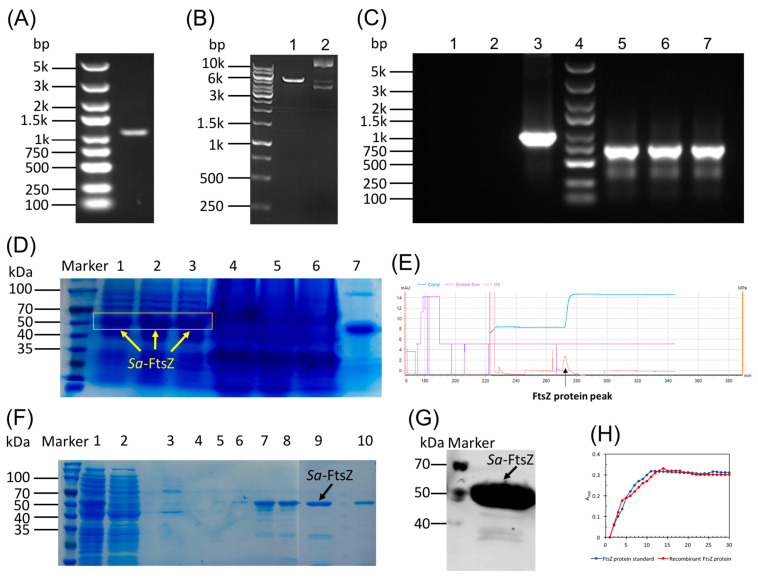
(**A**) Agarose gel electrophoresis of the target gene fragment obtained by PCR, with a gene size of 1213 bp. (**B**) Agarose gel electrophoresis of pET28a(+) plasmid after digestion with two restriction endonuclease enzymes, NheI and XhoI. Lane 1 represented the plasmid digestion product, and lane 2 showed the undigested plasmid. (**C**) Agarose gel electrophoresis of some fragments of the positive transformant of the recombinant clone obtained by PCR. Lane 1: negative control (ddH_2_O); lane 2: negative control (empty vector pET28a(+)); lane 3: positive control (GAPDH); lane 4: marker; lanes 5–7: transformant (pET28a(+)-*Sa*-FtsZ); lane 4: marker; lane 5: transformant (pET28a(+)-*Sa*-FtsZ). (**D**) The components of the supernatant and cell debris after centrifugation were analyzed by SDS-PAGE: 1~3 stood for the protein component of the supernatant; 4~5 represented the protein component of cell fragmentation; and 7 represented the standard protein FtsZ. (**E**) Protein purification curve by employing a protein purifier, and the FtsZ protein peak appeared at 270 min. (**F**) SDS-PAGE analysis of purified protein samples: 1 represented the protein sample before purification; 2 represented the penetrating fluid of the sample flowing through the column; 3, 4, 5, and 6 represented the protein samples eluted by eluent containing 5, 10, 25, and 50 mM imidazole, respectively; 7, 8, 9, and 10 represented the protein samples eluted by eluent containing 100 mM imidazole. (**G**) Identification of purified *Sa*-FtsZ protein by Western blot analysis. (**H**) Polymerization kinetics of FtsZ standard protein and recombinant FtsZ protein, determined by monitoring the absorbance at 340 nm (A_340_) within 30 min.

PCR was performed using the synthesized single DNA strand as a template in the presence of positive and negative primers. Ultimately, the length of the PCR product (*Sa*-FtsZ gene) was 1213 bp, which was identified by agarose gel electrophoresis (Figure 2A). Additionally, agarose gel electrophoresis of pET28a(+) plasmid digested with NheI and XhoI was displayed in Figure 2B, and verified that the fragment is correct. Furthermore, some fragments of the positive transformant of the recombinant clone were obtained. The size of the PCR product was 679 bp. Agarose gel electrophoresis result (Figure 2C) demonstrated that positive converters were correct. Additionally, the sequencing results were compared with the sequences of the *S. aureus* FtsZ gene (ID: EU914258.1) in the NCBI database, and the matching degree was 100%.

To determine whether the target protein was successfully expressed and its location of expression, the components of the supernatant and cell debris after centrifugation were analyzed by SDS-PAGE. As displayed in Figure 2D, both the supernatant and precipitate contained a variety of proteins. The supernatant included a protein with an approximate molecular weight of 55 kDa, framed with a yellow wire, corresponding to the FtsZ protein. This protein matched the size of the standard FtsZ protein, demonstrating the correctness of the expressed target protein. Nevertheless, the proteins in the cell fragments contain almost no target proteins. These results illustrated that the target protein was successfully expressed, and the expression position was outside the cell of the bacteria, that is, in the supernatant of the bacterial fermentation fluid.

Since the cell supernatant contained many miscellaneous proteins other than the FtsZ protein, it was necessary to purify the protein. The purification was achieved by employing the ÄKTA pure protein purifier, with a distinct peak appearing at 270 min (Figure 2E). Subsequent analyses via SDS-PAGE and Western blot (Figure 2F,G) confirmed the high purity of the isolated *Sa*-FtsZ protein. Imidazole buffers with concentrations of 5, 10, 25, and 50 mM could only elute heteroproteins, while eluent containing 100 mM imidazole can eluate the target protein, with the tenth tube displaying excellent purity. Consequently, we obtained pure FtsZ protein by employing gradient elution.

The purified protein was concentrated with the ultrafiltration tube and stored at −80 °C for later use. Then, the protein concentration was determined by the Bradford protein quantitative method. Finally, we calculated a protein concentration of 1.46 mg/mL, with the aid of the standard curve of BSA.

Next, the FtsZ polymerization activity assay was performed on the purified protein to evaluate its biological activity. As shown in Figure 2H, the FtsZ polymerization activity of the recombinant protein *Sa*-FtsZ expressed in this study was basically consistent with that of the FtsZ standard protein. This result demonstrated that the recombinant *Sa*-FtsZ protein we expressed retained good biological activity.

### 2.4. FtsZ Polymerization Assay

**Figure 3:**
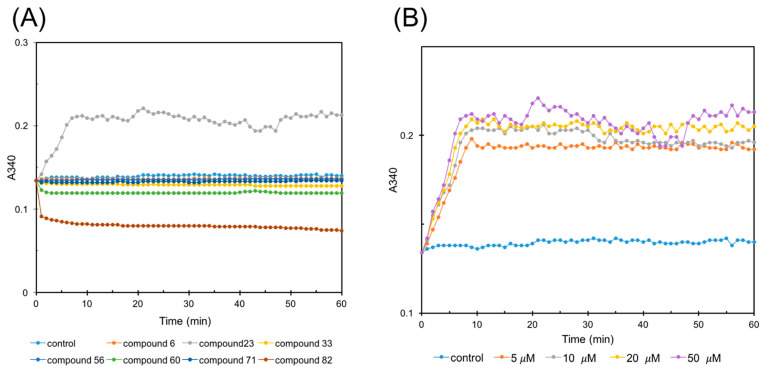
(**A**) Effect of 50 μM compounds (DMSO, **6**, **23, 33, 56**, **60**, **71,** and **82**) on FtsZ assembly in vitro; (**B**) effect of different concentrations (5, 10, 20, and 50 μM) of compound **23** on FtsZ assembly in vitro. The polymerization of GTP-induced FtsZ was monitored as the absorbance at 340 nm. The experiment was performed in triplicate, and the average absorbance is shown.

Given that compound **82** is a well-documented FtsZ inhibitor [22], renowned for its potent inhibition of FtsZ polymerization, it served as a valuable positive control in subsequent experimental endeavors. The FtsZ polymerization assay was meticulously conducted, and the outcomes were elegantly presented in Figure 3A. Notably, compound **23** stood out for its remarkable promoting effect on FtsZ polymerization, particularly evident during the initial 10 min of the reaction, when compared to the DMSO control. In addition, compound **82**, as anticipated, demonstrated robust inhibitory activity against FtsZ polymerization, reinforcing its established properties. In addition, compound **60** also exhibited apparent inhibitory tendencies towards FtsZ polymerization, suggesting a potential role as an inhibitor. Moreover, compounds **33** and **56** had minimal impact on the dynamics of FtsZ polymerization, indicating either weak or neutral effects. The experiment was performed in triplicate, and the average absorbance is displayed.

To delve deeper into the effects of compound **23**, its influence on FtsZ polymerization was assessed across various concentrations (5, 10, 20, and 50 μM). As depicted in Figure 3B, a striking trend emerged: compound **23**, at concentrations ranging from 5 to 50 μM, markedly accelerated FtsZ polymerization, particularly during the initial 10 min of the reaction. Notably, the promoting effect of compound **23** was concentration-dependent, with higher concentrations eliciting a more pronounced enhancement of FtsZ polymerization. This observation underscores the potential of compound **23** as a modulator of FtsZ dynamics, with important implications for the regulation of cell division processes.

### 2.5. Sedimentation Assay

**Figure 4:**
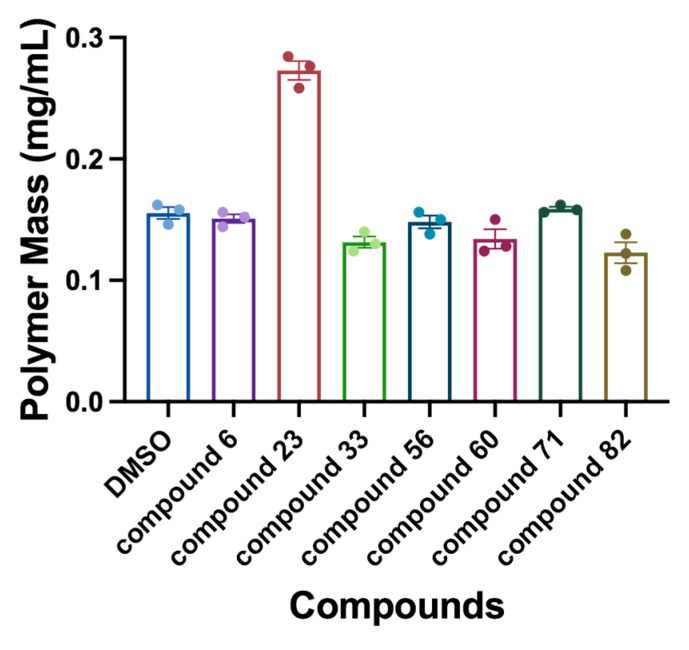
Sedimentation analysis for the detection of FtsZ polymerization amount. The experiment was performed in triplicate, and the average absorbance is shown.

The results of the sedimentation assay, presented in Figure 4, are closely consistent with the results of the FtsZ polymerization assay. Notably, treatment with compound **23** markedly enhanced protein sedimentation compared to the control group, indicative of a robust promotion of FtsZ polymerization. In addition, compounds **33**, **60**, and **82** exhibited varying degrees of inhibition on FtsZ polymerization, while compounds **6**, **56**, and **71** had negligible effects.

### 2.6. Affinity Assay by SPR Technology

**Figure 5:**
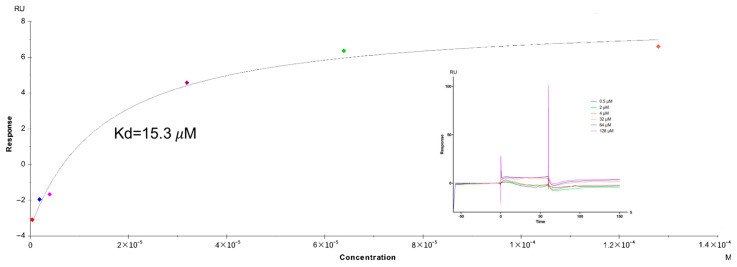
The affinity test result of compound **23** on FtsZ protein chip (KD = 15.3 μM). Colored dots represent the response values exhibited at different concentrations.

The FtsZ protein was successfully coupled to the four pathways of the CM5 chip, achieving a coupling amount of 12,564.5 RU. To further investigate the specificity of the chip, the affinity between compound **23** and FtsZ was rigorously tested. Affinity analysis results were depicted in Table 2, which unveiled that the equilibrium dissociation constant (KD) of compound **23** binding to FtsZ protein was 15.3 μM. The sensing signal diagram of compound **23** binding to FtsZ protein and the fitting curve are shown in Figure 5. The results illustrated that compound **23** had a potent affinity with the FtsZ protein, which aligns seamlessly with the previous results, demonstrating that compound **23** remarkably promoted FtsZ polymerization.

### 2.7. Phase-Contrast Microscopy

**Figure 6:**
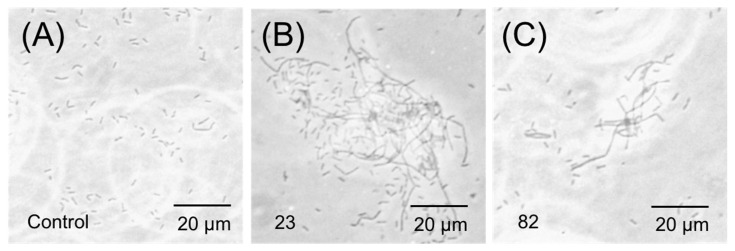
Inhibition of cell division by compounds **23** and **82**. Cells of *B. subtilis* were grown in the absence (**A**) and presence of 64 μM of **23** (**B**) and **82** (**C**).

Given the remarkable effects of compounds **23** and **82** on FtsZ polymerization, the morphology of *B. subtilis* treated with them was observed and studied. Phase-contrast microscopy revealed intriguing alterations in bacterial morphology upon treatment with these compounds (Figure 6). In the parallel control group without drug treatment, almost no linearized cells were detected. In contrast, both compounds induced varying degrees of elongation in the rod-like structures of *B. subtilis*, with compound **23** exhibiting the most pronounced effect. This observation is in harmony with the conclusion that these compounds influence FtsZ polymerization (promote or inhibit), thereby blocking bacterial division, providing additional validation for their efficacy in modulating FtsZ polymerization.

### 2.8. Visualization of Z-Ring in Bacterial Cells

**Figure 7:**
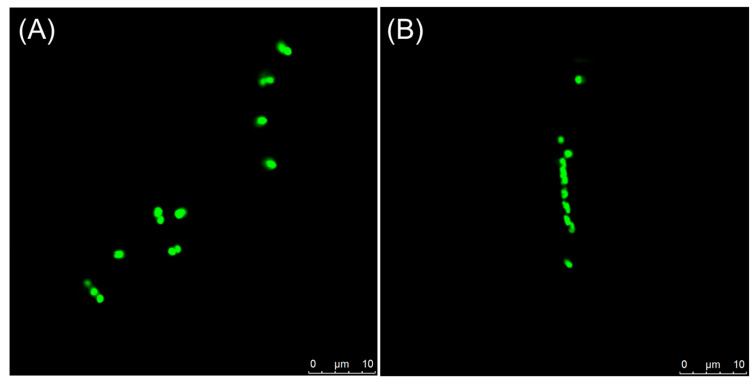
The visualization of the Z-ring in bacterial cells. Cells of *E. Coli* BL21 (DE3) were, respectively, grown with 1% DMSO (**A**) and 64 μM compound **23** (**B**).

Previous studies have shown that some FtsZ inhibitors can perturb FtsZ function to inhibit the formation of the Z-ring. Confocal imaging of live *E. Coli* cells with GFP-tagged FtsZ was performed under 1% DMSO and 64 μM concentration of compound **23**. In the control group, the normal pattern of FtsZ localization with bands of FtsZ-tagged GFP fluorescence (Z-ring) was observed at the middle of the cell (Figure 7A). Treatment with 64 μM of compound **23** disturbed the Z-ring assembly to inhibit cell division and result in cell elongation (Figure 7B), probably indicating that FtsZ is a target of this compound.

### 2.9. In Vivo Antibacterial Activity Testing

**Figure 8:**
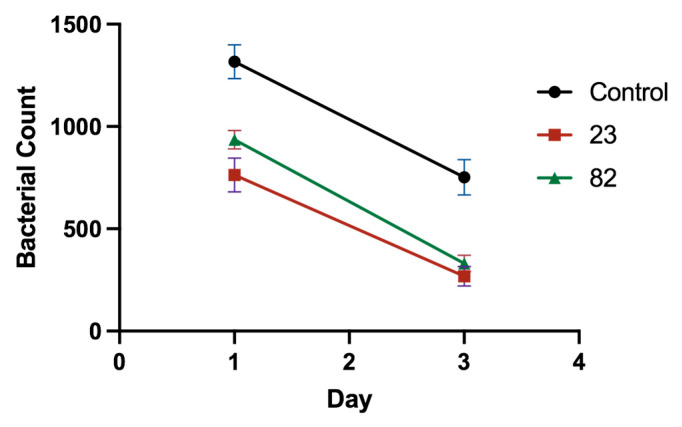
Bacterial counts in the peritoneal fluid of mice. Control: inoculated with bacteria; **23**: inoculated with bacteria and treated with compound **23**; **82**: inoculated with bacteria and treated with compound **82**. On the first and third days after administration of bacteria and test drugs, 2 mL of sterile physiological saline was injected into the abdominal cavity of the mice, and the abdominal fluid was extracted after gently diluting for 2 min under sterile conditions. The experiment was performed in triplicate, and the average absorbance is shown.

The results of the bacterial count of peritoneal fluid are depicted in Figure 8. As shown in the figure, the bacterial count of the peritoneal cavity of mice in both the treatment group and the non-treatment group exhibited a downward trajectory over time, suggesting an inherent capacity for self-recovery among the animals. Notably, the bacterial count in the abdominal cavity of mice in the untreated group was notably elevated, whereas it was substantially mitigated in those administered compounds **23** and **82**. Furthermore, the bacterial burden in the group treated with compound **23** was lower than that of the **82** treatment group, underscoring the superior therapeutic efficacy of compound **23** compared to **82**.

Throughout the seven-day observation period, the clinical presentations of the infected mice in each group were meticulously recorded. The untreated mice displayed characteristic symptoms such as erect hair, anorexia, diminished food intake, pronounced lethargy, dyspnea, and general discomfort. In contrast, mice treated with compounds **23** and **82** exhibited vitality, maintained a healthy appetite, and their respiratory and cardiac rates remained within normal limits, as compared to the control group. Additionally, the therapeutic outcomes in the **23** treatment group surpassed those of the **82** treatment group.

Upon necropsy on the seventh day, a macroscopic examination of the vital organs revealed stark differences between the treatment and untreated groups. The untreated mice exhibited substantial mucus accumulation in their chest and abdominal cavities, accompanied by yellowish-white fecal soiling around the anus and a fishy-smelling, yellowish, and watery intestinal content. Additionally, their livers and spleens were enlarged and congested, with grayish necrosis evident on the liver surface and in cross-sections. The lungs were swollen, congested, and edematous. Conversely, the organs of the treated mice displayed signs of recovery or had reverted to their normal physiological states.

### 2.10. Antiproliferative Activities

**Figure 9:**
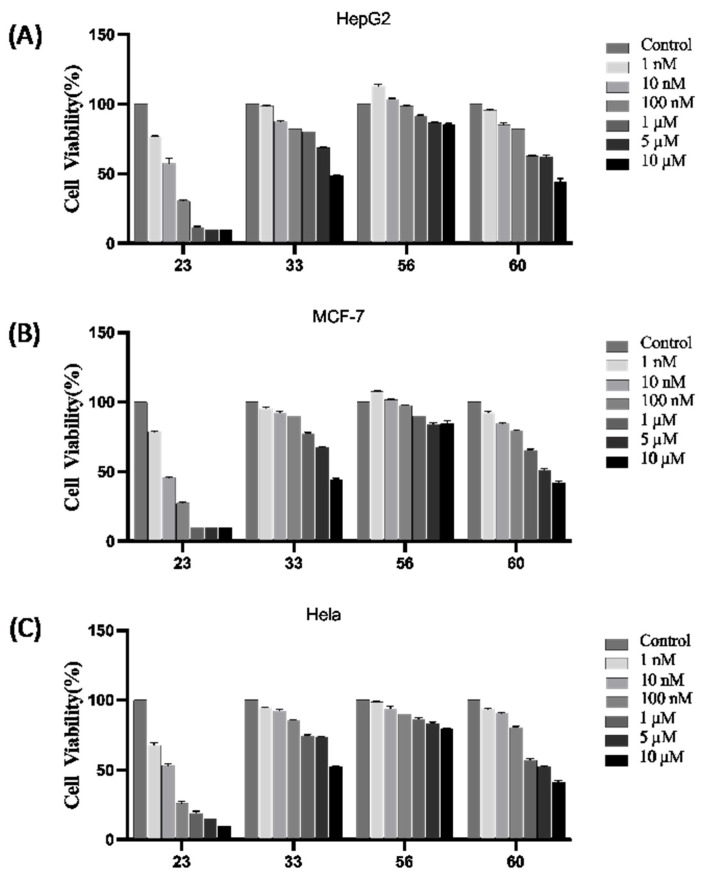
The results of antiproliferative activity against HepG2 (**A**), MCF-7 (**B**), and Hela (**C**) cells of the compounds (**23**, **33**, **56,** and **60**) determined by the CCK8 method.

Given that the compounds in question are anticancer agents specifically targeting tubulin, we have undertaken a rigorous revalidation of their anticancer efficacy, focusing on compounds **23**, **33**, **56,** and **60**. As evident from the results presented in Figure 9, all of these compounds possessed antiproliferative activities, which demonstrated that we have obtained some compounds with both anticancer and antibacterial activities. In particular, compound **23** showed the most potent anticancer activity. The cell viability after treatment with compound **23** at a concentration of 100 nM was only 30.5%, 27.5% and 26% for HepG2, MCF-7, and Hela, respectively, and the cell viability at a concentration of 1 μM was only 11.5%, 10% and 19% for HepG2, MCF-7, and Hela, respectively.

Further, we assessed the capacity of these compounds to impede tubulin polymerization, as shown in Table 3, and the anti-tubulin polymerization activity of the compounds is consistent with the anticancer cell activity. Notably, compound **23** manifested stronger antitubulin activities (IC_50_ = 0.02 μM) than colchicine (IC_50_ = 3.08 μM) and CA-4 (IC_50_ = 2.44 μM). Additionally, compound **60** also represented potent inhibitory activity against tubulin polymerization, with an IC_50_ of 5.62 μM.

### 2.11. Cytotoxicity

**Figure 10:**
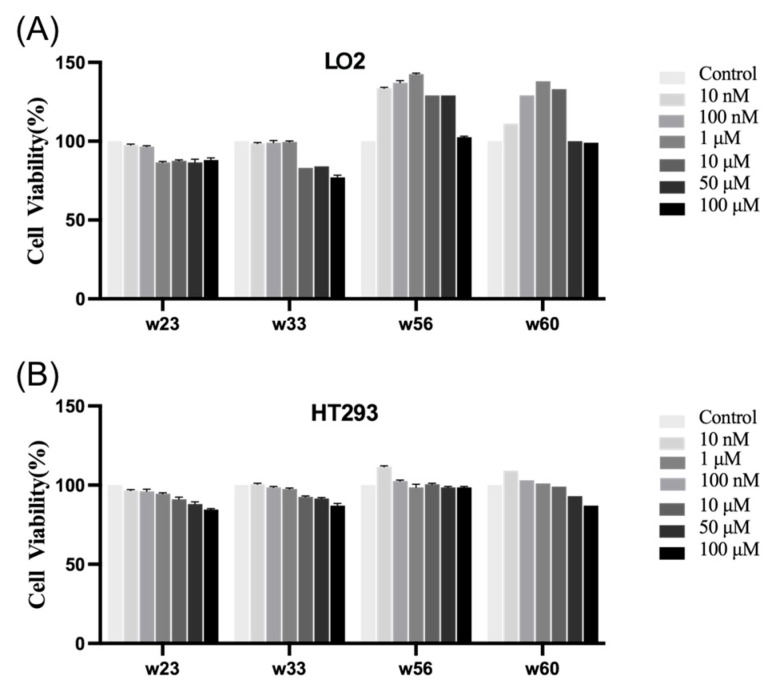
The changes in cell viability of LO2 (**A**) and 293T (**B**) cells after co-culture with the compound for 12 h. Results shown are the mean ± SD of at least three separate determinations.

In addition, to ascertain the safety profile of compounds **23**, **33**, **56**, and **60** towards eukaryotic mammalian cells, we estimated their toxicity against LO2 and 293T cells using the CCK8 assay. The results, presented in Figure 10, revealed the changes in cell vitality after 12 h of exposure to these compounds. At the highest concentration tested (100 μM), compounds **23**, **33**, **56**, and **60** induced mild cytotoxicity, reducing viability to 81%, 65%, 99%, and 90%, respectively, for LO2 cells and 79%, 80%, 98%, and 89%, respectively, for HT293 cells. The selective cytotoxicity of the compound likely stems from its specific targeting of microtubules, which are critical for cancer cell proliferation due to their high mitotic activity. Normal cells, with lower division rates and more robust regulatory mechanisms, are less affected by this mechanism, resulting in reduced toxicity. The results suggested their potential suitability as antibacterial and anticancer agents for human use without posing undue harm to host cells.

### 2.12. Molecular Docking Study

**Figure 11:**
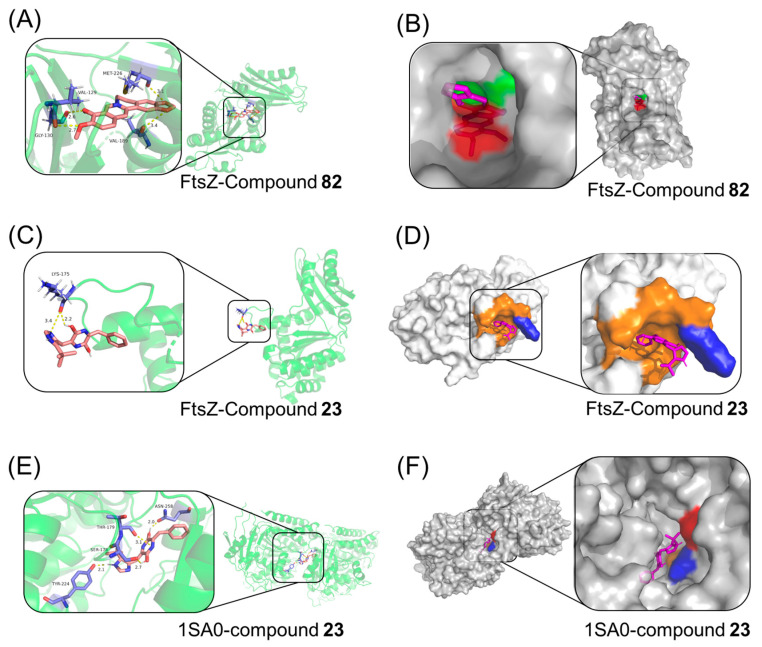
Docking modes of compound with protein. (**A**,**C**) A 3D molecular docking modeling of compound **82**/**32** with *S.aureus* FtsZ (PDB code: 4DXD). (**B**,**D**) The surface model structure to display the interaction between compound **82**/**32** and the targeted protein *S.aureus* FtsZ. (**E**) A 3D molecular docking modeling of compound **23** with tubulin (PDB code: 1SA0). (**F**) The surface model structure to display the interaction between compound **23** and the targeted protein, tubulin.

Docking is an effective and reliable approach to simulate the probable binding mode of ligands and proteins [23]. To visualize the possible binding model of interactions between a protein and small molecules, the molecular docking techniques were employed. The above experiments proved that compound **23,** a drug with both anticancer and antibacterial activity, so the molecular dockings of compound **23** with both tubulin and FtsZ were simulated by computer. Since compound **82** only possessed antibacterial activity, the molecular docking of compound **82** with FtsZ was performed. The involved amino acid residues were labeled. As can be seen from the resulting figure (Figure 11A,B), compound **82** bound to the GTP binding pocket of FtsZ, which contained VAL 129, GLY 130, VAL 189, and MET 226, with a binding energy estimated at −21.3 kcal/mol. The oxygen of the two methoxys in the benzene ring formed hydrogen bonds with VAL 129 and GLY 130 at distances of 2.7 and 2.6 Å, respectively. In addition, the other two hydrogen bonds connected the amino acid VAL 189 and MET 226 to the two oxygens on the five-membered ring with 3.4 and 3.1 Å, respectively.

As depicted in Figure 11C,D, compound **23** binds to the T7 loop binding pocket of FtsZ, and they were closely bonded by two hydrogen bonds, accompanied by a binding energy estimated at −27.6 kcal/mol. The hydrogen bond distance between N at the 2-position of the five-membered ring and LYS175 was 3.4 Å; the other hydrogen bond formed between the phenyl hydroxyl group on the pyrazine ring and LYS175 at a distance of 2.2 Å. The predicted binding energy between **23** and FtsZ is lower than that of **82** and FtsZ, indicating a stronger binding ability between **23** and FtsZ, which also corresponds to better antibacterial activity of **23**.

Figure 11E,F expressed the binding mode of compound **23** interacting with tubulin (binding energy estimated at −34.1 kcal/mol), and the docking results revealed that four amino acids SER178, THR179, TYR224, and ASN258 located in the binding pocket of protein played vital roles in the conformation with compound **23**, which were stabilized by four hydrogen bonds. One hydrogen bond (2.7 Å) connected amino acid SER178 to hydroxyl group on the pyrazine ring with intermediated position with styrene; the second hydrogen bond with 2.0 Å was formed between ASN258 and the other hydroxyl group on the pyrazine ring; the third hydrogen bond (3.1 Å) was formed between THR179 and the nitrogen on the pyrazine ring with intermediated position with styrene; and the fourth hydrogen bond with 2.1 Å connected amino acid TYR224 to the hydrogen attached to nitrogen on the five element heterocyclic ring.

## 3. Discussion

At present, there are very few reports on dual-active anticancer and antibacterial drugs, and only a few reports, such as natural peptide melittin [24], plant extracts silybin [25,26], and jujube flavonoids [27,28,29], have not been widely used due to their respective shortcomings, and there is a lack of clear structure–activity relationship research.

To address this challenge, we analyzed the characteristics of cancer cells and bacteria and found that they have homologous proteins, namely tubulin in cancer cells and FtsZ in bacteria. The literature has reported that some drugs have both microtubule protein and Ftsz protein inhibitory activity. Therefore, drugs with these characteristics can be used as both anticancer and antibacterial drugs. This study constructed a microtubule inhibitor compound library containing 196 compounds, which were then validated the anticancer activity and detected antibacterial activity. Meanwhile, the antibacterial mechanism of the screened compounds was investigated. Through this study, we have obtained six microtubule inhibitors (**6**, **23**, **33**, **56**, **60**, and **71**) with excellent antibacterial activity. In addition, compounds **23**, **33**, **56**, and **60** had confirmed interactions with FtsZ, and compound **23** exhibited the most potent FtsZ affinity. In this study, the screened several compounds with both antibacterial activities were screened to enrich the types of antibacterial drugs. Simultaneously, compound **23** was a concentration-dependent FtsZ inhibitor with strong affinity, which has certain significance for subsequent drug development and further expansion. Additionally, we analyzed compounds with good antibacterial activity and found that their structures contain N or S heterocycles, indicating that compounds with such structures are more likely to exhibit anticancer and antibacterial activities. This discovery provides an important theoretical basis for the selection of such compounds based on experimental and computational studies in the future. In the near future, we believe that the research of anticancer and antibacterial dual-active drugs will become an indispensable class of drugs for cancer patients. Furthermore, FtsZ is highly conserved in its natural state, with a low tendency towards intrinsic functional resistance mutations, though inhibitor-specific mutations may occur under inhibitor pressure (e.g., PC190723) [30].

## 4. Materials and Methods

### 4.1. Materials

The number of compounds in the library is 196, and some of them were purchased from companies such as Target Molecule Corp. (Boston, MA, USA), Aladdin Reagent Co., Ltd. (Shanghai, China), MedChemExpress Biotechnology Company (Shanghai, China), et al.; some were accumulated from previous experiments; and the remaining were obtained through chemical synthesis. The detailed information pertaining to compounds in this extensive library is comprehensively outlined in Table S1 (Supplementary Materials file). Four bacterial strains were utilized in the experiments. *E. coli* ATCC 8739, *S. aureus* ATCC 6538, and *P. aeruginosa* DSM 939 were supplied by BeNa Culture Collection; *B. subtilis* CICC 21164 was provided by the School of Food Science, China Agricultural University. A DNA recycling kit was procured from Tiangen Biochemical Technology Co., Ltd. (Beijing, China). The restriction endonucleases (NheI, XhoI) and T4 DNA ligase were purchased from Takara Bio Inc. (Shiga, Japan). LB and SOB broth medium were supplied by Shanghai Boguang Biotechnology Co., Ltd. (Shanghai, China). *E. coli* BL21 (DE3) and Ampicillin were provided by Sangon Biotech Co., Ltd. (Shanghai, China). HepG2, MCF-7, Hela, LO2, and 293T cells were preserved by Nanjing Agricultural University. Ni-NTA His-Tag purification agarose was purchased from MedChemExpress Biotechnology Co., Ltd. (Shanghai, China). Coomassie Bright Blue staining kit and Bradford protein quantitative kit were provided by Shanghai Beyotime Biotechnology Co., Ltd. (Shanghai, China). The FtsZ protein standard is purchased from Cytoskeleton Inc. (Denver, CO, USA).

### 4.2. Methods

#### 4.2.1. Determination of Minimum Inhibitory Concentration (MIC)

MIC assays were carried out by employing the broth microdilution method in accordance with Clinical and Laboratory Standards Institute (CLSI) guidelines [21,31]. *E. coli* BNCC337004, *S. aureus* BNCC336423, *P. aeruginosa* BNCC337099, and *B. subtilis* CICC 21164 were applied for the MIC detection. Mueller–Hinton broth (MHB) was used in bacterial culture, and log-phase bacteria were diluted into a 0.5 McFarland Standard suspension, which was then diluted 1000 times again. Subsequently, 100 μL of the obtained bacterial suspension was placed into each well of 96-well microtiter plates, along with 2-fold serial dilutions of compounds (512, 256, 128, 64, 32, 16, 8, 4, 2, 1, 0.5, 0 μM). The microtiter plates were incubated aerobically for 18 h at 37 °C. The MIC was defined as the lowest drug concentration at which no visible turbidity was observed. All experiments are conducted in three parallel.

#### 4.2.2. Time–Killing Curve

*S. aureus* BNCC336423 was employed for the time–killing curve study [32]. Approximately 2 × 10^5^ CFU/mL bacteria in MH medium were prepared, and 2 mL of the initial culture was placed into each tube, which contained compounds (**6**, **23**, **33**, **56**, **60**, **71,** or **82**) at final concentrations ranging from 0× to 8× the MIC. Meanwhile, DMSO with an equivalent volume was added to the vehicle control tube. The cultures were then subjected to shaking incubation at 37 °C. Subsequently, cultures were taken out at predetermined time intervals (0, 1, 2, 4, 6, 8, and 24 h), centrifuged at 16,000×g for 3 min, and diluted to an appropriate multiple. These diluted samples were then plated onto Tryptic Soy Agar (TSA) plates for enumeration. Following a 24 h incubation period at 37 °C, colonies on the TSA plates were counted for the CFU/mL, thereby providing insights into the time-dependent bactericidal activity of the tested compounds.

#### 4.2.3. Sa-FtsZ Expression and Purification

*S. aureus* FtsZ gene (GenBank: EU914258.1) retrieved from the NCBI database was synthesized by designing *NheI* and *XhoI* cleavage sites at both ends of the gene. Afterwards, the whole gene sequence was obtained and synthesized by Shanghai Genechem Co., Ltd. (Shanghai, China). With the assistance of the cleavage sites (NheI and XhoI), the obtained gene fragment was subsequently inserted into the pET28a(+) plasmid, resulting in the construction of the recombinant plasmid pET28a(+)-*Sa*-FtsZ. Later, pET28a(+)-*Sa*-FtsZ was transformed into *E.coli* BL21(DE3) competent cells, which would be spread on LB solid medium containing 100 µg/mL ampicillin, and cultured overnight at 37 °C. Afterwards, a single colony was picked and seeded into LB liquid medium containing 100 µg/mL ampicillin. When OD = 0.4, 1 mmol/L IPTG was added. After induction at 30 °C for 5 h, the expression product was collected, which was then purified by applying the ÄKTA pure 25 L protein purification instrument chromatography purification system. Since the recombinant protein was labeled with his, Ni-NTA His-Tag Purification Agarose (MedChemExpress Biotechnology Co., Ltd., Monmouth Junction, NJ, USA) was employed for the purification. The purity of the protein was assessed using SDS-PAGE and Western blot, while the protein concentration was determined with a Bradford quantitative assay kit. Also, the FtsZ polymerization activity assay was performed.

#### 4.2.4. SDS-PAGE and Western Blot

Finally, 10% SDS-PAGE and Western blot were used to detect the purified protein. Pre-stained protein marker (Amersham Biosciences UK, Ltd., Buckinghamshire, UK) was used in SDS-PAGE and Western blot. For SDS-PAGE, 10% PAGE gels were employed and stained with Coomassie Brilliant Blue, followed by destaining and gel imaging. For Western blot, gels were blotted against a nitrocellulose membrane (Hybond-ECLTM, Amersham Biosciences UK, Ltd.). After membrane transfer, membrane was blocked with 5% skimmed milk at room temperature for 1 h, then anti-His Tag Mouse Monoclonal Antibody (Shanghai Biyuntian Biological Co., Ltd., Shanghai, China) was used at 1/1000 dilution to incubate the membrane at 4 °C overnight, which was subsequently incubated in IRDye 800CW goat anti-mouse IgG(H + L) (LI-COR, Lincoln, NE, USA) for 1 h. Western blot detection was performed by employing the Odyssey imaging system according to the protocol recommended by the manufacturer.

#### 4.2.5. FtsZ Polymerization Assay

The effect of compounds on *S. aureus* FtsZ polymerization was monitored by employing a microtiter plate-based spectrophotometric assay method, and the reactions were conducted in flat-bottomed 96-well microtiter plates. The degree of FtsZ polymerization was reflected by the corresponding changes in absorbance at 340 nm (A_340_). An amount of 5 µM FtsZ was combined with compounds **6**, **23**, **33**, **56**, **60**, **71,** and **82** (50 µM) in 100 µL of reaction solution, which includes 50 mM Tris-HCl (pH 7.4), 10 mM magnesium acetate, and 50 mM KCl. In addition, different concentrations (5, 10, 20, and 50 µM) of compound **23** were also tested for the effect on *S. aureus* FtsZ polymerization. The polymerization reaction began immediately once 4 mM GTP was added, and then the polymerization was continuously monitored at 25 °C for 60 min by measuring A_340_ in BioTek-Synergy4 multifunctional enzyme-linked immunosorbent assay (ELISA) reader.

#### 4.2.6. Sedimentation Assay

Sedimentation assay was also conducted for the influence of compounds on FtsZ polymerization. FtsZ (5 μM) was polymerized in a buffer containing 25 mM Pipes buffer (pH 6.8) and 3 mM MgSO_4_ in the absence of DMSO or compounds (50 μM). After the addition of 4 mM GTP, the sample was instantly placed at 37 °C for 30 min. Then, the polymers were collected by centrifuging for 30 min at 25 °C. The protein concentration in the supernatant was determined using the Bradford quantification method, with bovine serum albumin (BSA) as the standard. The experiment was performed in triplicate, and the average absorbance is shown.

#### 4.2.7. Affinity Assay by SPR Technology

FtsZ Pre-Enrichment: FtsZ purified protein was dissolved in deionized water and prepared into a 1 g/L protein stock solution. Afterwards, protein concentration was diluted to 50 mg/L, respectively, using four different pH acetate buffer solutions (pH 4.0, 4.5, 5.0, 5.5). Subsequently, samples were injected, and the response values of protein FtsZ under different pH conditions were detected using the Biacore pre-enrichment system to determine the optimal protein coupling conditions.

FtsZ Coupling: Before coupling, the CM5 chip was first activated by EDC/NHS solution for 7 min; then, protein coupling was carried out, with a coupling time of 10 min and a protein flow rate of 10 μL/min; finally, the unreacted active sites on the chip were blocked by ethanolamine for 7 min.

The compounds were dissolved in DMSO, and then diluted to 50 μM with PBS (final concentration of DMSO was 5%). The mobile phase was a PBS solution containing 5% DMSO. The response value of the samples flowing through the surface of the FtsZ protein was analyzed by the Biacore T200 system. The monomer compounds with a higher response value to FtsZ (higher than or equal to the positive control) were screened as candidate compounds.

Kinetic Analysis: The process of kinetics is shown in Figure S1. The concentration of candidate compounds was diluted by gradient with a double ratio, with concentration ranging from 0 to 64 μM (the final concentration of DMSO was 5%). Then, the binding response value was obtained. Kinetic curves were drawn according to the dose–effect relationship between the response value and the concentration of candidate compounds, and the binding specificity of candidate compounds and FtsZ was determined according to the curve fitting. Thus, small molecular monomers that can bind specifically to the FtsZ protein were found.

#### 4.2.8. Phase-Contrast Microscopy

Cell Division Inhibitory Screening Assay Cell division inhibitory activity of compounds was conducted as below. Log-phase *B. subtilis* were adjusted to an OD_600_ of 0.06, and cultured in MHB at 37 °C for 4 h in the presence of DMSO (solvent control) or 64 μM compounds (**23** or **82**). Afterwards, 1 mL of the sample was centrifuged at 15,000× *g* for 3 min at room temperature, and the bacterial pellets were washed with 1 mL of PBS before re-centrifugation. The final bacterial pellets obtained were resuspended in 5 mL PBS. 20 μL of bacterial suspension was transferred to slides for microscopy. Cell morphology was estimated by using the phase-contrast method of Olympus IX71 microscopy (Olympus Corporation, Tokyo, Japan) with 400× magnification.

#### 4.2.9. Visualization of Z-Ring in Bacterial Cells

The assay was carried out using a strain of *E.coli* BL21 (DE3) containing an IPTG-inducible plasmid, which can produce the green fluorescence protein (GFP)-tagged FtsZ to make the Z-ring visible. An overnight culture of *E.coli* was replated into 1 mL fresh MHB medium and incubated at 37 °C. Until OD_600_ = 0.3, 40 μM IPTG (Isopropyl *β*-D-1-thiogalactopyranoside) was added. The cells were treated with 1% DMSO and 64 μM compounds **23**, respectively, for 4 h at 37 °C. After that, they were centrifuged and re-suspended in PBS, and then observed using the Leica SP-5 laser scanning confocal microscopy. Z-ring analysis was performed by employing LAS-AF-Lite software 2.6.0.

#### 4.2.10. In Vivo Antibacterial Activity Testing

SPF grade CD-1 male mice weighing 30 ± 3 g were purchased from Shanghai Slake Experimental Animal Co., Ltd. (Shanghai, China). In the experiment, bacterial mouse models were established to evaluate the in vivo antibacterial activities of compounds exhibiting promising in vitro antibacterial and FtsZ inhibitory properties. Initially, all mice were intraperitoneally injected with a 1/2 lethal dose (LD_50_) concentration of *E. coli*, with an injection volume of 0.01 mL/g body weight. Immediately following this, the mice were intraperitoneally injected with the test compounds. Each experimental group consisted of five mice, with each compound being tested at two distinct concentration gradients (determined based on the minimum inhibitory concentration, MIC). A separate group received physiological saline as a negative control. The subsequent assessment focused on evaluating the therapeutic potential of these drugs against bacterial infections. On the first and third days post-administration, 2 mL of sterile physiological saline was injected into the peritoneal cavity of each mouse. After a gentle two-minute dilution under sterile conditions, the abdominal fluid was extracted for bacterial enumeration using the plate counting method. Furthermore, the clinical manifestations of each mouse group, encompassing mental status, appetite, and other relevant parameters, were meticulously observed over a seven-day period. On the seventh day, the mice were euthanized, and a thorough macroscopic examination was conducted to assess the pathological changes in various vital organs.

#### 4.2.11. Antiproliferative Activities

Antiproliferative activities of compounds were detected by employing the CCK8 method. HepG2, MCF-7, and Hela cells were cultured to log phase in DMEM medium supplemented with 10% fetal bovine serum. Cells were diluted to 2 × 10^4^ cells/mL, and 100 µL of the obtained suspension was seeded in each well of the 96-well microplates, which were then cultured at 37 °C overnight. Subsequently, cells were treated with compounds at different concentrations. After co-culturing for 48 h, wells were washed with PBS three times and filled with 100 μL DMEM complete medium, which contained CCK8. After culturing for 2 h at 37 °C, the absorbance of measured and recorded on an ELISA reader (BioTek-Synergy 4, Winooski, VT, USA) at a test wavelength of 450 nm. Three replicate wells were employed for each drug concentration, and each assay was conducted at least three times.

#### 4.2.12. Effects of Tubulin Polymerization

In addition, the compounds (**23**, **33**, **56**, and **60**) with good antibacterial and anticancer activities were tested for tubulin polymerization inhibition activity according to the literature, as well as the positive control agents (colchicine and CA-4). Compounds with a certain concentration were pre-incubated with 10 μM bovine brain tubulin in glutamate buffer at 30 °C, and then cooled to 0 °C. The absorbance of 350 nm was immediately measured at 30 °C, as soon as the addition of 0.4 mM GTP. The absorbance would be detected continuously for 20 min. The IC_50_ was defined as the compound concentration that inhibited tubulin polymerization by 50% during 20 min.

#### 4.2.13. Cytotoxicity

The cytotoxic activity of compounds (**23**, **33, 56**, and **60**) in vitro was measured against the human normal hepatic cell (LO2), the human renal epithelial cell (293T), and the human macrophage by using the CCK8 method. The absorbance of 450 nm was measured in an ELISA reader (BioTek-Synergy 4, USA). Then, cell viability was calculated, and the corresponding images were drawn with GraphPad Prism 9.

#### 4.2.14. Molecular Docking Study

A molecular docking study was performed using the AutoDock 4.2 software package. The preparation of ligands and receptors, grid parameter settings, and docking analysis were conducted with AutoDock Tools 1.5.6 graphical interface.

Ligand Preparation: The initial 3D structures of the compounds were constructed and optimized by energy minimization in Chem3D 21.0 to obtain stable conformations. Import the optimized structures into AutoDock Tools and perform the following processing: add Gasteiger charges, detect rotatable keys (all rotatable keys are recognized and set as rotatable). Finally, the ligand was saved in pdbqt format.

Protein Receptor Preparation: The X-ray crystal structure (PDB code: 4DXD [33]) of the *S. aureus* FtsZ bound with 9PC402 and GDP was downloaded from the RCSB Protein Data Bank (PDB) (http://www.rcsb.org). Both the original ligand 9PC402 and all water molecules were then removed from the crystal structure 4DXD. Subsequently, polar hydrogen atoms were added to the protein, and Kollman combined atomic charges were assigned. All bonds of the receptor protein were considered rigid. The processed receptor was saved in pdbqt format.

Grid Parameter Settings: The search space for docking is defined through the grid module in AutoDock Tools. The center coordinates of the grid box are set to (X = −6.69, Y = 30.062, Z = 21.397). To ensure sufficient search space for the ligand, the grid box size was set to 60 × 60 × 60 grid points, with a grid point spacing of 0.375 Å. This means that the physical dimensions of the box are approximately 22.5 Å × 22.5 Å × 22.5 Å, sufficient to accommodate the ligand and allow for its free rotation and translation.

Docking Parameters: Use the Lamarckian genetic algorithm of AutoDock 4 for semi-flexible docking calculation. The key parameter settings were set as follows: runs—50, population size—150, maximum evolutionary generation—27,000, intersection rate—0.80, mutation rate—0.02, and local search frequency—0.06. The above parameters ensured sufficient sampling of ligand conformation and orientation within the binding pocket.

Result Analysis and Combination Mode Selection: After docking, all conformations generated from 50 runs were clustered with a root mean square deviation (RMSD) tolerance of 2.0 Å. The representative conformation from the cluster with the lowest binding free energy was selected as the predicted optimal binding mode for subsequent interaction visualization analysis using PyMol Version 3.1.6.1 (https://pymol.sourceforge.net/).

Verification of Docking Method (Re-Docking Experiment): To verify the reliability of the docking process and parameters used in this study, we conducted a re-docking experiment. The specific steps were as follows: We extracted the eutectic ligand 9PC402 from the 4DXD crystal structure, used the same protein preparation method, grid parameters, and docking parameters as described above, and re-docked it back into the binding pocket of the FtsZ protein. We calculated the RMSD between the optimal conformation obtained by re-docking and the original crystal structure conformation. The re-docking conformation was highly consistent with the experimental conformation, with an RMSD value of 0.92 Å (less than the recognized threshold of 2.0 Å). This result strongly demonstrates that the docking method used in this study can accurately reproduce the binding patterns observed in experiments, and therefore is reliable and applicable.

## 5. Conclusions

In conclusion, compounds with anticancer and antibacterial dual activity were screened from the constructed tubulin inhibitor compound library (196 compounds). Six compounds (**6**, **23**, **33**, **56**, **60,** and **71**) were obtained, which possessed both anticancer and antibacterial activities. Among the compounds that targeted tubulin, **23**, **33**, **56,** and **60** were proven to target FtsZ. Particularly, compound **23** exhibited the most potent FtsZ polymerization-promoting activity, and the promoting effect was concentration-dependent. In addition, in the SPR assay, compound **23** represented strong FtsZ affinity with KD = 15.3 μM. Moreover, in phase-contrast microscope experiments and Z-ring visualization experiments, the length of *Bacillus subtilis* and *E.coli* increased distinctly after treatment with the compound **23**, which further proved that compound **23** targets FtsZ. Further, molecular docking studies revealed that compound **23** and LYS175 of FtsZ were closely bonded by two hydrogen bonds. Through this series of experiments, the mechanism of compound **23** inhibiting bacterial division through the FtsZ protein was elucidated.

## Figures and Tables

**Table 1:** MIC of compounds against four bacteria.

Compounds	Structure	MIC (μM)			
		*S. aureus* (ATCC 6538)	*B. subtilis* (CICC 21164)	*E. coli* (ATCC 8739)	*P. aeruginosa* (DSM 939)
**6**	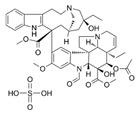	8	16	64	32
**23**	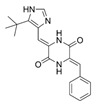	8	8	64	32
**33**	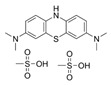	4	64	32	2
**56**	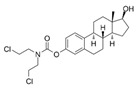	128	64	128	32
**60**	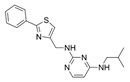	8	64	16	8
**71**	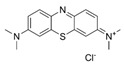	2	32	64	64
**82**	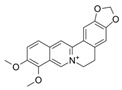	4	64	64	8

**Table 2:** The affinities of compound **23** with FtsZ.

Compound	KD (μM)	Rmax (RU)	Chi^2^
**23**	15.3	11.82	0.234

**Table 3:** In vitro inhibition of tubulin polymerization by compounds **23**, **33**, **56**, **60**, and control drugs (colchicine and CA-4).

Compounds	IC_50_^a^ ± SD ^b^ (μM)
	Tubulin
**23**	0.02 ± 0.31
**33**	74.46 ± 0.25
**56**	38.66 ± 0.28
**60**	5.62 ± 0.12
Colchicine ^c^	3.08 ± 0.13
CA-4 ^c^	2.44 ± 0.11

All experiments were independently performed at least three times. ^a^ Inhibition of tubulin polymerization. ^b^ SD: standard deviation. ^c^ Used as positive controls.

## Data Availability

The original contributions presented in this study are included in the article/Supplementary Materials. Further inquiries can be directed to the corresponding authors.

## Supplementary Materials

The following supporting information can be downloaded at: https://www.mdpi.com/article/10.3390/antibiotics14101014/s1, Table S1: Information of compounds in library; Figure S1. Schematic diagram of binding kinetics of compound to protein on chip.
