# Supplementary material for: Isolation of Dual-Active Drugs with Anticancer and Antibacterial Activities That Target Both Tubulin and FtsZ
Source: Antibiotics (Basel). 2025 Oct 13;14(10):1014. doi: 10.3390/antibiotics14101014 (PMC12561670; doi:10.3390/antibiotics14101014)
Supplement: Supplementary file 1 [file antibiotics-14-01014-s001.zip › antibiotics-3791415-supplementary.pdf]

Tables and Figures

Table S1. Information of compounds in library

Index	Compound Name	Structural Formula	Molecular Formula	Molecular Weight
1	Docetaxel trihydrate	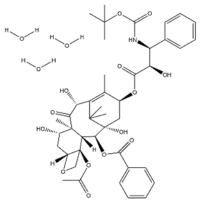	$C_{43}H_{59}NO_{17}$	861.95
2	Docetaxel	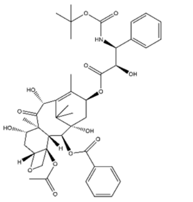	$C_{43}H_{53}NO_{14}$	807.88
3	Podofilox	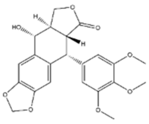	$C_{22}H_{22}O_{8}$	414.41
4	Albendazole	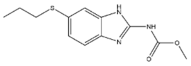	$C_{12}H_{15}N_{3}O_{2}S$	265.33
5	Mebendazole	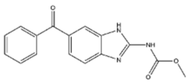	$C_{16}H_{13}N_{3}O_{3}$	295.3

Index	Compound Name	Structural Formula	Molecular Formula	Molecular Weight
6	Vincristine sulfate	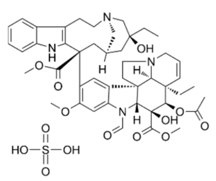	$C_{46}H_{58}N_4O_{14}S$	923.04
7	Vindoline	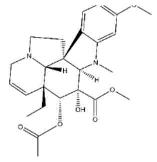	$C_{25}H_{32}N_2O_6$	456.54
8	4'-Demethylepipodophyllotoxin	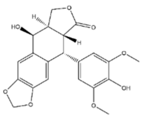	$C_{21}H_{20}O_8$	400.38
9	N-Phenylbenzylamine	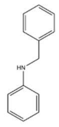	$C_{13}H_{13}N$	183.25
10	Oxfendazole	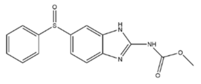	$C_{15}H_{13}N_3O_3S$	315.35
11	Parbendazole	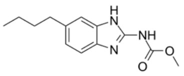	$C_{13}H_{17}N_3O_2$	247.29

Index	Compound Name	Structural Formula	Molecular Formula	Molecular Weight
12	Thiabendazole	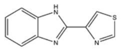	$C_{10}H_7N_3S$	201.25
13	Nocodazole	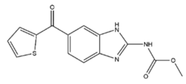	$C_{14}H_{11}N_3O_3S$	301.32
14	Vinblastine sulfate	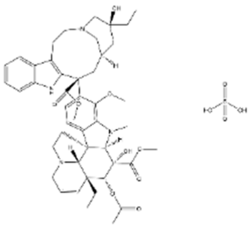	$C_{46}H_{58}N_4O_9 \cdot H_2SO_4$	909.06
15	Lexibulin	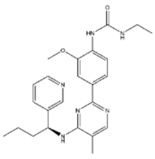	$C_{24}H_{30}N_6O_2$	434.53
16	Cabazitaxel	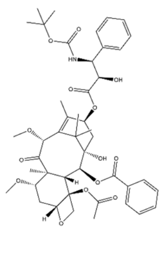	$C_{45}H_{57}NO_{14}$	835.93
17	10-Deacetyltaxol	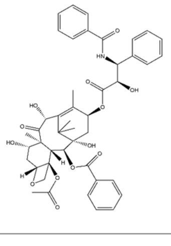	$C_{45}H_{49}NO_{13}$	811.87

Index	Compound Name	Structural Formula	Molecular Formula	Molecular Weight
18	Cephalomannine	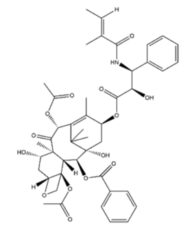	$C_{45}H_{53}NO_{14}$	831.91
19	CK-636	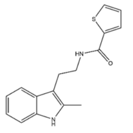	$C_{16}H_{16}N_2OS$	284.38
20	ABT-751	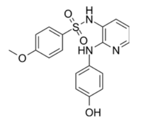	$C_{18}H_{17}N_3O_4S$	371.41
21	D-64131	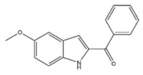	$C_{16}H_{13}NO_2$	251.28
22	INH1	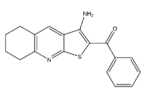	$C_{18}H_{16}N_2OS$	308.4
23	Plinabulin	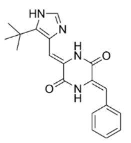	$C_{19}H_{20}N_4O_2$	336.39

Index	Compound Name	Structural Formula	Molecular Formula	Molecular Weight
24	7-epi-Taxol	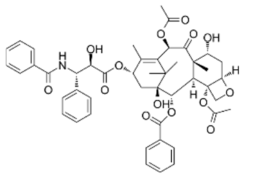	$C_{47}H_{51}NO_{14}$	853.91
25	Ansamitocin P 3'	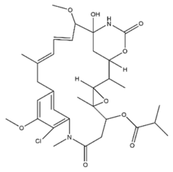	$C_{32}H_{43}ClN_2O_9$	635.14
26	Griseofulvin	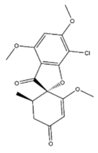	$C_{17}H_{17}ClO_6$	352.77
27	CW-069	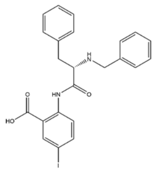	$C_{23}H_{21}IN_2O_3$	500.33
28	Vinorelbine	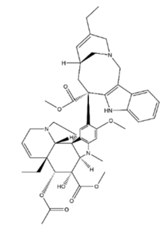	$C_{45}H_{54}N_4O_8$	778.93
29	Epothilone B	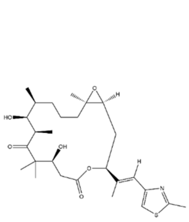	$C_{27}H_{41}NO_6S$	507.68

Index	Compound Name	Structural Formula	Molecular Formula	Molecular Weight
30	INH6	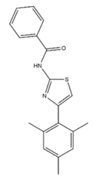	$C_{19}H_{18}N_2OS$	322.42
31	Tirbanibulin	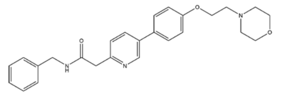	$C_{26}H_{29}N_3O_3$	431.53
32	7-epi-Taxol	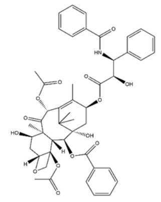	$C_{47}H_{51}NO_{14}$	853.91
33	Leucomethylene blue mesylate	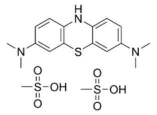	$C_{18}H_{27}N_3O_6S_3$	477.62
34	PE859	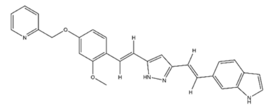	$C_{28}H_{24}N_4O_2$	448.52
35	Ansamitocin P 3’	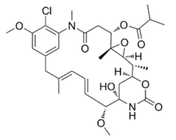	$C_{32}H_{43}ClN_2O_9$	635.14

Index	Compound Name	Structural Formula	Molecular Formula	Molecular Weight
36	Epothilone A	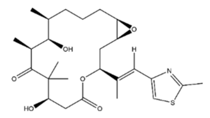	$C_{26}H_{39}NO_6S$	493.66
37	Ixabepilone	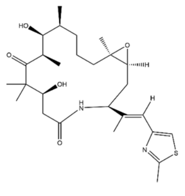	$C_{27}H_{42}N_2O_5S$	506.7
38	MMAD	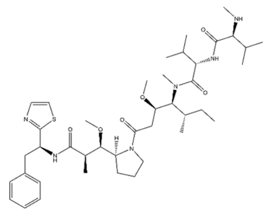	$C_{41}H_{66}N_6O_6S$	771.06
39	Benproperine phosphate	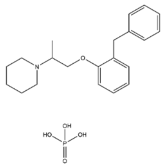	$C_{21}H_{30}NO_5P$	407.44
40	Albendazole sulfoxide	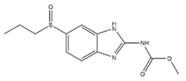	$C_{12}H_{15}N_3O_3S$	281.33
41	CK-869	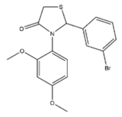	$C_{17}H_{16}BrNO_3S$	394.28

Index	Compound Name	Structural Formula	Molecular Formula	Molecular Weight
42	10-Deacetyl-7-xylosyl paclitaxel	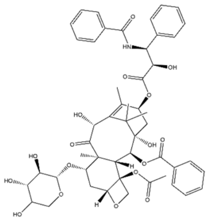	$C_{50}H_{57}NO_{17}$	943.98
43	Parbendazole	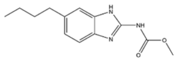	$C_{13}H_{17}N_3O_2$	247.29
44	SSE15206	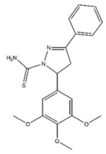	$C_{19}H_{21}N_3O_3S$	371.45
45	Podophyllotoxone	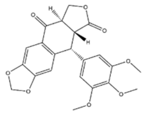	$C_{22}H_{20}O_8$	412.4
46	BRD9876	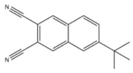	$C_{16}H_{14}N_2$	234.3
47	T-1101 tosylate	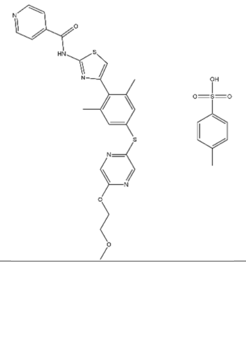	$C_{31}H_{31}N_5O_6S_3$	665.8

Index	Compound Name	Structural Formula	Molecular Formula	Molecular Weight
48	Verubulin hydrochloride	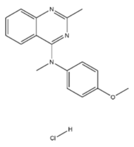	$C_{17}H_{18}ClN_3O$	315.8
49	Tubulin inhibitor 6	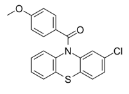	$C_{20}H_{14}ClNO_2S$	367.85
50	Indibulin	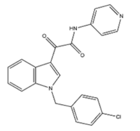	$C_{22}H_{16}ClN_3O_2$	389.83
51	McMMAF	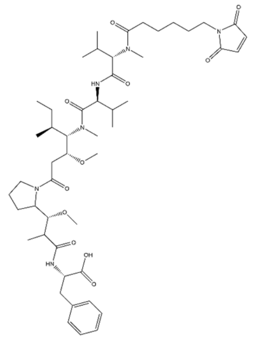	$C_{49}H_{76}N_6O_{11}$	925.16
52	Taccalonolide A	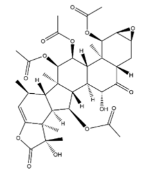	$C_{36}H_{46}O_{14}$	702.74
53	Maytansine	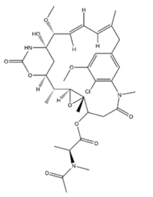	$C_{34}H_{46}ClN_3O_{10}$	692.2

Index	Compound Name	Structural Formula	Molecular Formula	Molecular Weight
54	Batabulin	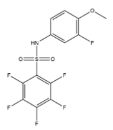	$C_{13}H_7F_6NO_3S$	371.26
55	BNC105	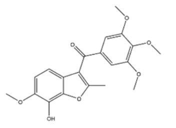	$C_{20}H_{20}O_7$	372.37
56	Estramustine	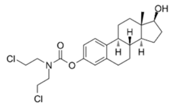	$C_{23}H_{31}Cl_2NO_3$	440.40
57	Tirbanibulin Mesylate	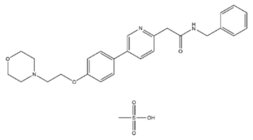	$C_{27}H_{33}N_3O_6S$	527.63
58	58	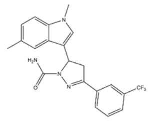	$C_{21}H_{19}F_3N_4O$	400.15
59	MAP4343	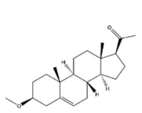	$C_{22}H_{34}O_2$	330.5

Index	Compound Name	Structural Formula	Molecular Formula	Molecular Weight
60	KHS 101	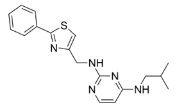	$C_{18}H_{21}N_5S$	339.46
61	Suprafenacine	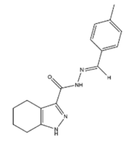	$C_{16}H_{18}N_4O$	282.34
62	Benproperine phosphate(39)	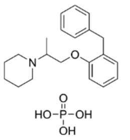	$C_{21}H_{30}NO_5P$	407.44
63	Crolibulin	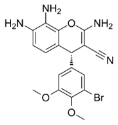	$C_{18}H_{17}BrN_4O_3$	417.26
64	Flubendazole	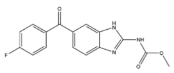	$C_{16}H_{12}FN_3O_3$	313.29
65	Paclitaxel	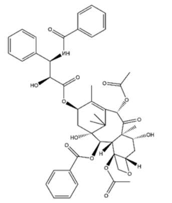	$C_{47}H_{51}NO_{14}$	853.92

Index	Compound Name	Structural Formula	Molecular Formula	Molecular Weight
66	2-Methoxyestradiol	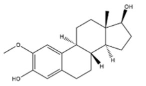	$C_{19}H_{26}O_3$	302.41
67	Combretastatin A4	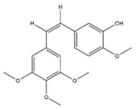	$C_{18}H_{20}O_5$	316.35
68	Monomethyl auristatin E	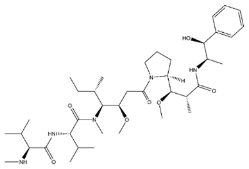	$C_{39}H_{67}N_5O_7$	717.98
69	Colchicine	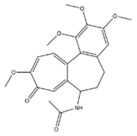	$C_{22}H_{25}NO_6$	399.44
70	MMAF	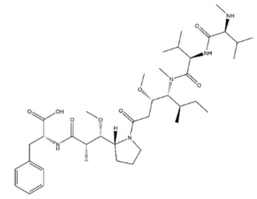	$C_{39}H_{65}N_5O_8$	731.98
71	Methylene Blue	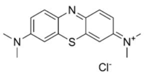	$C_{16}H_{18}ClN_3S$	319.85

Index	Compound Name	Structural Formula	Molecular Formula	Molecular Weight
72	Deoxypodophyllotoxin	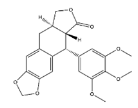	$C_{22}H_{22}O_7$	398.41
73	Myoseverin	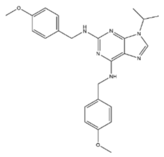	$C_{24}H_{28}N_6O_2$	432.57
74	Maytansinol	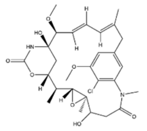	$C_{28}H_{37}ClN_2O_8$	565.06
75	CK-666	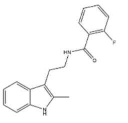	$C_{18}H_{17}FN_2O$	296.34
76	T807	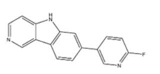	$C_{16}H_{10}FN_3$	263.27
77	Colcemid	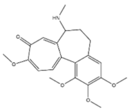	$C_{21}H_{25}NO_5$	371.43

Index	Compound Name	Structural Formula	Molecular Formula	Molecular Weight
78	Entasobulin	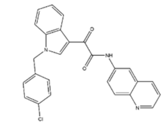	$C_{26}H_{18}ClN_3O_2$	439.89
79	Auristatin E	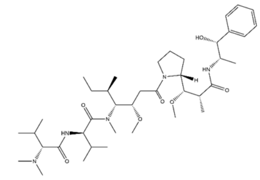	$C_{40}H_{69}N_5O_7$	732.01
80	Fosbretabulin Disodium	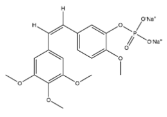	$C_{18}H_{19}O_8P \cdot 2Na$	440.29
81	Curcumin	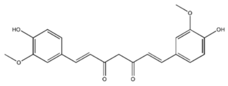	$C_{21}H_{20}O_6$	368.38
82	Berberine	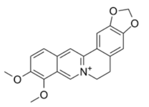	$C_{20}H_{18}NO_4^+$	336.36
83-110		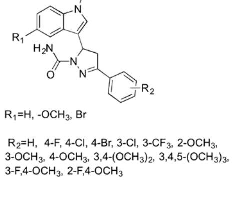		

Index	Compound Name	Structural Formula	Molecular Formula	Molecular Weight
111-134		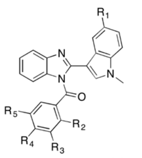<br>R_1, R_2, R_3=H, -Br, -OCH_3<br>R_4, R_5=H, -OCH_3		
135-152		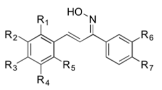<br>R_1, R_2, R_3, R_4, R_5=H, -OCH_3<br>R_6=H, OCH_3, Br, Cl, NH_2, NO_2<br>R_7=H, OCH_3, F, Cl, Br, -OC_2H_5		
153-174		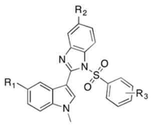<br>R_1=H, Br, -OCH_3<br>R_2=H, CH_3, Br<br>R_7=H, 4-CH_3, 4-OCH_3, 4-Cl, 4-Br, 4-CF_3, 2,5-$(OCH_3)_2$, 3,4-$(OCH_3)_2$		
175-196		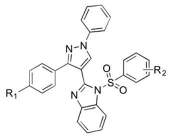<br>R_1=CH_3, F, -CF_3<br>R_2=H, 4-F, 4-Cl, 4-Br, 4-CF_3, 4-CH_3, 4-OCH_3, 2,5-$(CH_3)_2$, 4-NO_2		

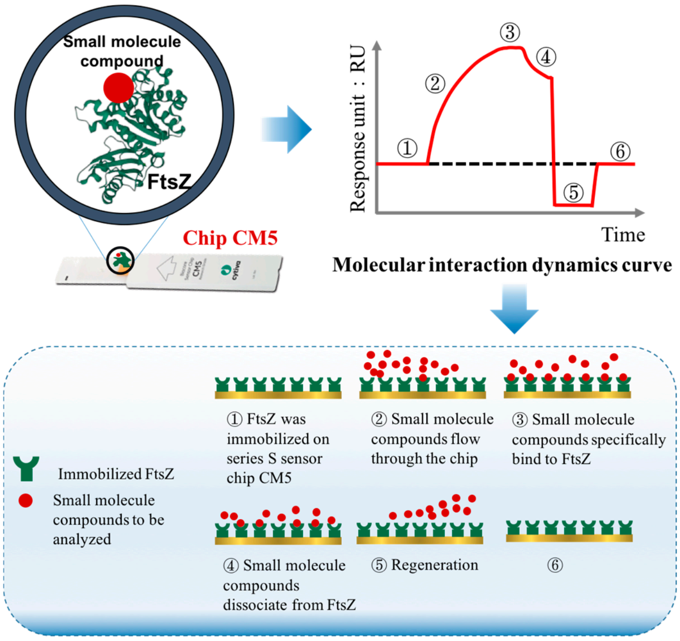

Fig.S1. Schematic diagram of binding kinetics of compound to protein on chip
